# Willingness and hesitancy towards the governmental free human papillomavirus vaccination among parents of eligible adolescent girls in Shenzhen, Southern China

**DOI:** 10.1186/s12905-024-03083-2

**Published:** 2024-04-23

**Authors:** Dadong Wu, Peiyi Liu, He Wang, Wenwen Wan, Yueyun Wang

**Affiliations:** 1 Shenzhen Maternity and Child Healthcare Hospital, No. 2004 Hongli Road, Shenzhen, China; 2 Shenzhen Key Laboratory of Modern Toxicology, Shenzhen Medical Key Discipline of Health Toxicology, Shenzhen Center for Disease Control and Prevention, Shenzhen, China

**Keywords:** Human papillomavirus, Vaccination, Adolescent, Willingness, Vaccine hesitancy, Influencing factor

## Abstract

**Background:**

Since 2020, China has actively promoted HPV vaccination for eligible adolescent girls through various pilot programmes. This study investigated parental willingness and hesitancy towards the government-sponsored, free human papillomavirus (HPV) vaccination for eligible adolescent girls in Shenzhen, Southern China.

**Methods:**

From June to August 2022, a cross-sectional survey was conducted with parents of girls entering Grade 7, employing an adapted Vaccine Hesitancy Scale to assess vaccine hesitancy and logistic regression to identify factors influencing willingness to accept the free domestic vaccines.

**Results:**

Although only 3.4% of the 2856 respondents had their daughters vaccinated against HPV prior to the survey, 91.7% were willing to utilise the governmental vaccination services. Parents with children in public schools (*χ*^*2*^ = 20.08, *p* < 0.001), those with more secure medical insurance (*χ*^*2*^ = 4.97, *p* = 0.026), and parents who had received an HPV vaccine themselves (*χ*^*2*^ = 28.829, *p* < 0.001) showed more reluctance towards the free vaccines. Vaccine hesitancy was presented in a mere 2.1% but was a significant predictor of vaccine refusal, even after adjusting for multiple factors (adjusted OR = 15.98, 95% CI: 9.06, 28.20). Notably, about four-fifths of parents of unvaccinated daughters harboured concerns about the safety and efficacy of the domestic vaccine.

**Conclusions:**

Although parents show a strong inclination to utilise the government vaccination services, their vaccine hesitancy, driven by safety concerns and a preference for imported vaccines, remains a significant barrier for rolling out vaccination coverage. This study highlights the need for multifaceted intervention strategies that address these issues to enhance HPV vaccine uptake effectively.

**Supplementary Information:**

The online version contains supplementary material available at 10.1186/s12905-024-03083-2.

## Background

Cervical cancer ranks the fourth most prevalent gynaecologic cancer worldwide [1]. Remarkably, China contributes to approximately 18% of the global incidence and 17% of the associated mortality [2]. According to the National Central Cancer Registry, in 2015, China witnessed 115,000 new cases of cervical cancer and 34,000 related deaths [3]. Considering that 95% of cervical cancer cases result from the persistent infection of high-risk human papillomavirus (HPV) [4] and given the proven efficacy of HPV vaccines in preventing HPV-related diseases [5–8], the urgency of scaling up HPV vaccination as a primary measure to curb the disease’s mortality becomes evident. In August 2020, the World Health Organization (WHO) launched its “Global Strategy to Accelerate the Elimination of Cervical Cancer as a Public Health Problem”, aiming to vaccinate 90% of girls with an HPV vaccine by the age of 15 by 2030 [9]. As a testament of the global commitment, by March 2022, 117 countries (60% of WHO Member States) had already integrated HPV vaccines into their national immunisation programmes [10]. Yet, despite the introduction of HPV vaccines in mainland China in 2016, the vaccination coverage among girls aged 9–15 remains at merely 2.2% [11].

Recognising the global impetus, China initiated HPV vaccination pilots in a number of cities from 2020, offering school-aged girls with either free vaccines or vaccination compensation [12]. In a significant step, WHO prequalified China’s first domestically produced HPV vaccine, Cecolin™, in October 2021. Not only is this vaccine safe and highly efficacious against HPV 16 and HPV 18 [7, 8], but it also proves more cost-effective than imported vaccines [13], amplifying its potential in both global and national cervical cancer prevention campaigns. Capitalising on this development, Shenzhen, a major city in Guangdong Province, Southern China, rolled out a free HPV vaccination programme in October 2022, targeting all girls in Grade 7 and below the age of 14 with the domestic bivalent vaccine [14]. However, uncertainties loom regarding the willingness and attitudes towards this government initiative among parents of eligible girls, whose ethical considerations and cultural values can significantly affect decisions related to adolescent vaccinations [15]. The willingness of parents/guardians to accept the governmental vaccination services can be influenced by various factors. Recent studies have shown that parents of adolescent girls in China with higher levels of education and income, better knowledge of HPV vaccine, and personal vaccination experiences were more inclined to vaccinate their daughters with an HPV vaccine [16, 17]. Vaccine hesitancy, a recurring challenge with multiple vaccines [18], also plays a pivotal role. As indicated by Wei et al., 53.9% of parents of female secondary school students in China exhibited hesitancy towards HPV vaccines [19]. Furthermore, the school-based nature of many vaccination programmes pose potential barriers, including perceptions of HPV vaccine being unsuitable for school-aged girls, conflicts to cultural norms, disagreement from schools, as well as the overshadowing of other health education priorities [20].

To guide the promotion and implementation of the governmental free HPV vaccination in Shenzhen, a survey was undertaken from June to August 2022. The survey aimed to assess the willingness and attitudes of parents of eligible girls towards the initiative, just before their daughters transitioned to Grade 7. More specifically, it sought to identify factors influencing parents’ willingness to vaccinate their children. By understanding these determinants, the study endeavoured to provide practical insights for enhancing the programme’s advocacy and execution.

## Methods

### Study design and participants

A cross-sectional study was conducted between June and August 2022 to assess the willingness and attitudes towards the governmental free HPV vaccination among parents of eligible girls. The sample size was determined to be 4000, using PASS 15.0 software (NCSS). A stratified cluster sampling method was utilised, wherein schools with Grade 6 classes were randomly selected from each of the 10 districts in Shenzhen. The selection was based on the total number of schools in each district, employing computer-generated random numbers. Ultimately, 32 schools were included in the analysis to satisfy the sample size requirements. Parents from these selected schools were all considered eligible for the study if they had a daughter entering Grade 7 in September 2022 and consented to participate in the survey. Those who declined participation were excluded.

### Data collection and questionnaire

Data for this study was collected using the Chinese online survey platform, “Questionnaire Star” (https://www.wjx.cn/). Participants could access the electronic questionnaire either by scanning a provided Quick Response code or clicking a link generated by the platform. The questionnaire, comprising 15 items in Chinese, was collaboratively designed by cervical cancer prevention programme managers, epidemiologists, academics, and representatives from the Municipal Education Bureau, drawing from existing literature and comprehensive discussions. The items covered sociodemographic information, awareness and knowledge of HPV and HPV vaccines, vaccine hesitancy, willingness to receive the vaccination services, as well as parents’ and children’s medical insurance details. An English version of the questionnaire is provided in the electronic supplementary material. In Shenzhen, adult medical insurance primarily consists of Shenzhen Employee Medical Insurance and commercial medical insurance. Shenzhen Employee Medical Insurance, provided by employers, is categorised into three tiers based on the premium paid and the benefits received. Tier 1 allows direct payment for children’s vaccine costs when the account balance exceeds 5000 Chinese yuan, whilst Tier 2 or Tier 3 do not cover children’s vaccine costs. The coverage of children’s vaccines by commercial medical insurance depends on the specific terms of the insurance contract. Both adults and children not covered by any type of medical insurance were categorised as “out-of-pocket payment” in this study. The dissemination of the questionnaire to parents of eligible girls in the selected schools was facilitated by the Municipal Education Bureau. This approach was chosen to maximise outreach and participation through the established networks of the Education Bureau.

### Assessment of HPV vaccine hesitancy

Parental HPV vaccine hesitancy was evaluated using the Vaccine Hesitancy Scale (VHS), originally developed by Larson et al. based on global evidence on vaccine hesitancy and a review of various survey tools [21]. Modified versions of the VHS have been utilised in assessing HPV vaccine hesitancy in several countries, including Canada [22], the United Kingdom [23], the United States [24], and Guatemala [25]. In this study, the 10-item VHS was tailored to the specific context of HPV vaccination in China and reduced to 8 items (Table 1). Two items, “Childhood vaccines are important for my child’s health” and “My child/children does or do not need vaccines for diseases that are not common anymore”, were excluded as they were either redundant or not directly relevant to HPV vaccination. The remaining 8 items were translated into Chinese and incorporated into the survey questionnaire.

**Table 1:** Adapted Vaccine Hesitancy Scale for assessing parental hesitancy towards the governmental free HPV vaccination in Shenzhen

No.	Item
1	Getting the HPV vaccine is a good way to protect my daughter from cervical cancer.
2	Having my daughter vaccinated against HPV is important for the health of others.
3	The HPV vaccine offered by the government programme is beneficial.
4	The information I receive about the HPV vaccine from the vaccination programme is reliable.
5	Generally, I do what healthcare providers recommend about vaccines for my daughter.
6	New vaccines carry more risks than older vaccines.
7	Domestic vaccines are less effective and safe compared to imported vaccines.
8	I am concerned about serious side effects of the HPV vaccine.

Aligning with previous literature [26], a 4-point Likert scale without a neutral response option was adopted. Negatively worded items were reverse-coded. For scoring purposes, and to ensure compatibility with certain established scoring systems, responses for each item were mapped onto a 5-point scale (strongly agree = 1, somewhat agree = 2, somewhat disagree = 4, strongly disagree = 5), ensuring that higher values consistently indicated greater hesitancy. The average score across the 8 items was computed. Referring to previous studies [24, 27], the midpoint “3” was used as the cutting score for identification of HPV vaccine hesitancy.

### Statistical analysis

Statistical analyses were performed using the Statistical Analysis Software (SAS) (SAS Institute Inc.). Descriptive analysis was used to present the sociodemographic characteristics and vaccination experiences of the participants. The differences in these baseline characteristics between parents willing and those unwilling to obtain the free HPV vaccines for their daughters were assessed using Chi-square test or Fisher’s exact test, as appropriate, with a *p*-value of less than 0.05 set as the threshold for statistical significance. The impact of vaccine hesitancy on parental acceptance of the governmental vaccination services was determined using multivariable logistic regression, suitable to the binary outcome of parental willingness. Variables showing statistical significance in the preliminary comparison between willing and unwilling groups were included in the final model. These variables included school classification (public or private), awareness of HPV and HPV vaccines (yes or no), mother’s HPV vaccination status (yes or no), and specific types of medical insurance for both parents and daughters. Each included medical insurance type was coded as separate categorical variables (yes or no). Vaccine hesitancy was categorised as yes (VHS score greater than 3) or no (VHS equal or below 3). The variance inflation factor (VIF) was calculated to assess multicollinearity among variables in the final model, with a VIF value less than 10 considered acceptable to mitigate concerns about multicollinearity. A two-sided *p*-value of less than 0.05 was deemed statistically significant.

## Results

### Baseline characteristics of the respondents

About 4000 online surveys were distributed to parents of eligible girls from June to August 2022, of which 2856 (71.4%) were completed and deemed eligible for analysis. The distribution, facilitated by the Municipal Education Bureau, supported broad participation but limited access to the precise number of distributed surveys, as well as the exact count of non-responders and refusals. This constraint has affected the capacity to report these specific figures. The sociodemographic characteristics and vaccination experiences of the respondents are summarised in Table 2. Among the 2856 respondents, 91.2% were mothers, and 99.4% were Chinese nationals. Additionally, 50.6% had prior knowledge of HPV and HPV vaccines, 1.6% had direct relatives diagnosed with cervical cancer. Of note, 20.7% reported that the girls’ mothers had previously been vaccinated against HPV, and 3.4% indicated that their daughters had already received an HPV vaccine. For the analysis assessing the impact of vaccine hesitancy on parental acceptance of the governmental vaccination services, only data from parents whose daughters had not previously received any HPV vaccine were included.

**Table 2:** Baseline characteristics of the respondents (*n* = 2856)

Variables		n	Ratio
Relationship	Mother	2606	91.2%
Nationality	Chinese	2840	99.4%
School classification	Public school	1905	66.7%
Medical insurance type of parents	Tier 1 Shenzhen Employee Medical Insurance	1817	63.6%
	Tier 2 or Tier 3 Shenzhen Employee Medical Insurance	840	29.4%
	Cross-regional medical insurance	94	3.2%
	Commercial medical insurance	124	4.3%
	Out-of-pocket payment	275	9.6%
	Others	28	0.9%
Medical insurance type of girls	Shenzhen Children’s Medical Insurance	2585	90.5%
	Shenzhen Family Medical Insurance	107	3.7%
	Cross-regional medical insurance	74	2.5%
	Commercial medical insurance	123	4.3%
	Out-of-pocket payment	217	7.5%
	Others	28	0.9%
Direct relatives diagnosed with cervical cancer	Yes	47	1.6%
Awareness of HPV and HPV vaccines	Yes	1447	50.6%
Mother vaccinated against HPV	Yes	591	20.7%
Daughter vaccinated against HPV	Yes	96	3.4%

### Parents’ willingness to get the free HPV vaccines for their daughters

Out of the 2760 respondents whose daughters had not received any HPV vaccine prior to the survey, a substantial majority (91.7%) expressed willingness to utilise the governmental vaccination services. Table 3 displays the factors associated with this willingness. Compared to parents of children in private schools, those with children in public schools exhibited less inclination to obtain the free vaccines for their daughters (*χ*^*2*^ = 20.08, *p* < 0.001). There were statistically significant differences in the types of medical insurance held by both parents and adolescents. Specifically, parents with Tier 1 Shenzhen Employee Medical Insurance were more inclined to reject the free vaccines (*χ*^*2*^ = 34.04, *p* < 0.001). Conversely, parents and girls without any medical insurance (out-of-pocket payment) demonstrated greater willingness to accept the free vaccines (*χ*^*2*^ = 4.97, *p* = 0.026 and *χ*^*2*^ = 4.515, *p* = 0.034, respectively). Interestingly, mothers who themselves had been vaccinated against HPV showed less adherence to the governmental free HPV vaccination (*χ*^*2*^ = 28.829, *p* < 0.001).

**Table 3:** Willingness of parents of unvaccinated girls to accept the free HPV vaccines (*n* = 2760)

Variables		Willing to accept (*n* = 2531)	Unwilling to accept (*n* = 229)	χ^2^	p
Relationship	Mother	2308 (91.2%)	217 (94.8%)	3.447	0.070
Nationality	Chinese	2516 (99.4)	229 (100%)	1.365	0.270
School classification	Public school	1644 (65.0%)	189 (82.5%)	28.08	< 0.001
Medical insurance type of parents	Tier 1 Shenzhen Employee Medical Insurance	1565 (61.8%)	186 (81.2%)	34.04	< 0.001
	Tier 2 or Tier 3 Shenzhen Employee Medical Insurance	787 (31.1%)	34 (14.8%)	26.53	< 0.001
	Commercial medical insurance	107 (4.2%)	16 (7.0%)	3.76	0.053
	Out-of-pocket payment	246 (9.7%)	12 (5.2%)	4.97	0.026
	Others	23 (0.9%)	2 (0.9%)	0.003	0.283*
Medical insurance type of girls	Shenzhen Children’s Medical Insurance	2288 (90.4%)	213 (93.0%)	1.688	0.194
	Shenzhen Family Medical Insurance	84 (3.3%)	19 (8.3%)	14.49	0.0001
	Commercial medical insurance	106 (4.2%)	13 (5.7%)	1.128	0.288
	Out-of-pocket payment	197 (7.8%)	9 (3.9%)	4.515	0.034
	Others	27 (1.1%)	0 (0.0%)	2.467	0.163*
Direct relatives diagnosed with cervical cancer	Yes	43 (1.7%)	1 (0.4%)	2.24	0.276
Awareness of HPV and HPV vaccines	Yes	1284 (50.7%)	101 (44.1%)	3.688	0.055
Mother vaccinated against HPV	Yes	446 (17.6%)	79 (34.5%)	28.829	< 0.0001

* Analysed by Fisher’s exact test

### Impact of vaccine hesitancy on parents’ willingness to vaccinate their daughters

**Fig. 1:**
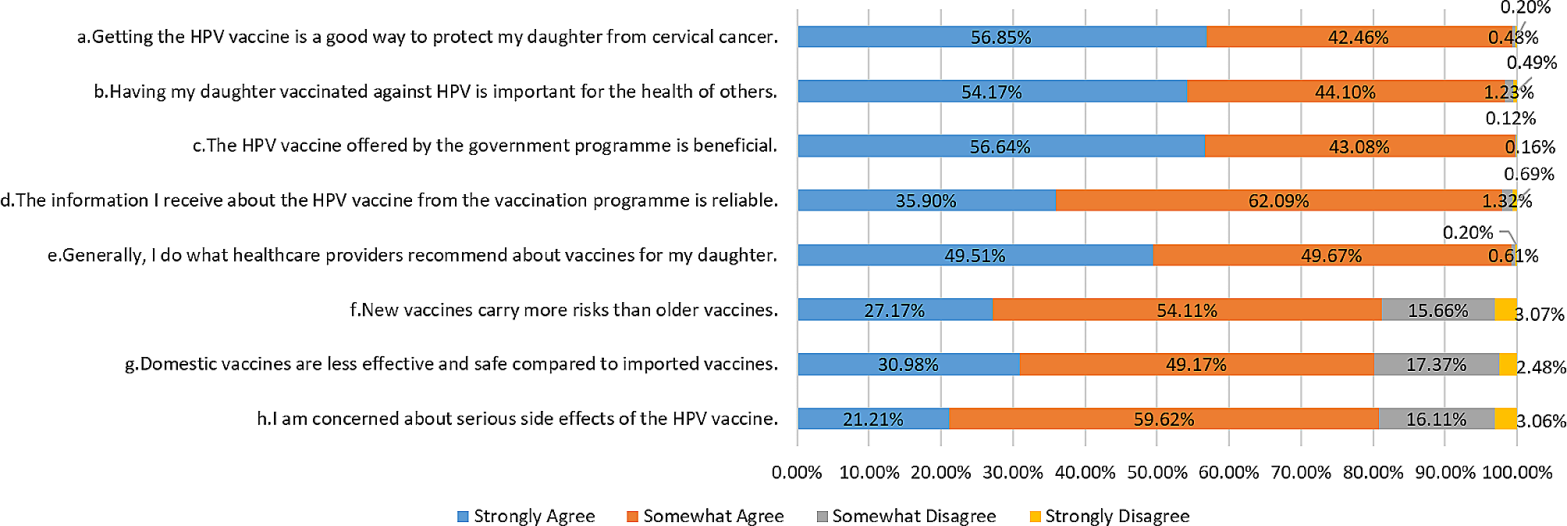
Responses of parents of unvaccinated girls to the adapted 8-item Vaccine Hesitancy Scale

The mean and median scores on the adapted 8-item VHS for parents of unvaccinated girls were identically low, at 2.00. Notably, only 59 respondents (2.1%) demonstrated a VHS score above 3, indicating hesitancy towards the HPV vaccine. Parents’ responses to the 8 VHS items are presented in Fig. 1. A vast majority (over 99%) expressed belief in the benefits of the government-provided vaccine, recognising its potential to protect their daughters from cervical cancer and contribute to public health. Most parents tended to adhere to healthcare providers’ recommendations regarding vaccines for their children, with only a small fraction (2.0%) expressing distrust in the information obtained from the HPV vaccination programme. However, concerns were also prevalent. A significant 80.8% of respondents harboured worries about serious side effects of the HPV vaccine, and 80.2% believed that imported vaccines are safer and more effective than domestic vaccines. Additionally, 81.3% concurred that new vaccines carry more risks than traditional ones.

Despite the low prevalence of vaccine hesitancy, its impact on parents’ willingness to utilise the governmental vaccination services was further analysed. As shown in Table 4, the crude logistic regression model revealed that hesitant parents were significantly more likely to refuse the free HPV vaccines (unadjusted OR = 13.99, 95% CI: 8.23, 23.80). Even after adjusting for nationality, awareness of HPV and HPV vaccines, school classification (public or private), medical insurance types (parents’ and daughters’), and mothers’ vaccination experiences, vaccine hesitancy remained a significant predictor of parental refusal to vaccinate their daughters against HPV (adjusted OR = 15.98, 95% CI: 9.06, 28.20).

**Table 4:** Logistic regression analysis of the impact of vaccine hesitancy on parents’ willingness to get the free HPV vaccines for their daughters

Models	Variables	Estimated value	Standard error	Wald x^2^	p	OR (95% CI)	VIF
Crude model	Vaccine hesitancy	2.64	0.27	94.81	< 0.0001	13.99 (8.23, 23.80)	
Adjusted model	Vaccine hesitancy	2.77	0.29	91.48	< 0.0001	15.98 (9.06, 28.20)	1.02
	School classification	-0.55	0.21	6.94	0.008	0.58 (0.38, 0.87)	1.32
	Mother’s awareness of HPV and HPV vaccines	0.38	0.15	6.21	0.013	1.46 (1.08, 1.97)	1.05
	Mother’s HPV vaccination status	-1.02	0.16	40.29	< 0.0001	0.36 (0.26, 0.50)	1.05
	Medical insurance type of parents: Tier 1 Shenzhen Employee Medical Insurance	0.014	0.38	0.0013	0.973	1.01 (0.48,2.13)	4.63
	Medical insurance type of parents: Tier 2 or Tier 3 Shenzhen Employee Medical Insurance	-0.411	0.42	0.94	0.333	0.66 (0.29, 1.52)	3.74
	Medical insurance type of parents: Commercial medical insurance	-0.591	0.37	2.54	0.113	0.55 (0.27, 1.15)	1.19
	Medical insurance type of parents: Out-of-pocket payment	-1.21	0.78	2.38	0.123	0.30 (0.07, 1.39)	1.79
	Medical insurance type of girls: Shenzhen Family Medical Insurance	0.84	0.28	8.88	0.003	2.31 (1.33, 3.99)	1.02
	Medical insurance type of girls: Out-of-pocket payment	-0.053	0.41	0.018	0.893	0.95 (0.43, 2.11)	1.31

## Discussion

Since 2020, China has been actively promoting HPV vaccination for eligible adolescent girls through various pilot programmes [12]. This survey, conducted just before the launch of Shenzhen’s municipal government-funded vaccination initiative, has been instrumental in raising parental awareness and refining implementation strategies. The findings revealed that only 3.4% of adolescent girls entering Grade 7 had already received an HPV vaccine in Shenzhen, which is consistent with the low coverage reported in studies from other regions [11, 13]. Remarkably, an overwhelming 91.7% of parents of unvaccinated girls expressed willingness to utilise the governmental free vaccination services. This inclination significantly exceeds the 52.9% willingness rate reported in a recent cross-sectional study of 5215 parents across four provinces in China [17]. Such a stark contrast may be attributed to the effectiveness of the voluntary school-based vaccination programme in boosting vaccination uptake in Shenzhen. This programme focused on promoting HPV vaccine awareness and access rather than administering vaccines at schools, a strategy that likely boosted willingness by increasing accessibility without tying vaccination to school attendance [14]. Supporting this notion, Lee and colleagues have demonstrated that a Home-School-Doctor model can significantly enhance adherence to HPV vaccination among both adolescent girls and their parents in Hong Kong, even when payment is required [28].

This study observed that parents and children with more secure medical insurance were more inclined to refuse the free HPV vaccines. Similarly, parents of children in public schools and those who had been vaccinated against HPV themselves showed less willingness to utilise the governmental vaccination services. These findings contrast with existing literature, which suggests that higher income levels and prior knowledge of HPV correlate with increased adherence to vaccination programmes in China [16, 17]. A plausible explanation is that individuals with greater financial resources and more knowledge about HPV may have access to alternative preventive measures, leading them to perceive less need for publicly funded health interventions and potentially impacting the overall infection load. Additionally, parents who had been vaccinated themselves may have preferred self-paid imported vaccines, especially considering that China’s first domestically produced HPV vaccine was prequalified in 2021. Their willingness to pay for HPV vaccines, influenced by factors such as vaccine valency and origin (imported versus domestic) [29], suggests a preference that might extend to their daughters’ vaccination choices. These trends underscore the importance of tailored communication strategies in future HPV vaccination campaigns, ensuring that messages resonate with diverse socio-economic groups and address specific concerns about vaccine necessity and trustworthiness, thereby enhancing overall vaccine uptake.

In this study, only 2.1% of parents whose daughters were unvaccinated against HPV had a VHS score greater than 3, a figure lower than those reported in Western countries [22–24]. Several factors may contribute to this difference. Firstly, the adaptation of the VHS for this study involved the removal of two items, which could potentially influence the overall hesitancy scoring. Additionally, the elimination of the neutral response option may have impacted the average scoring, potentially reducing reported hesitancy levels. Moreover, cultural and contextual factors in China, including public trust in healthcare policies and government-sponsored health initiatives, may inherently contribute to lower vaccine hesitancy among parents compared to other countries. These aspects warrant further exploration to fully understand their impact on vaccine hesitancy.

Despite the seemingly low rate, vaccine hesitancy was identified as a significant predictor of parental refusal to accept the free HPV vaccines, even after adjusting for various factors. Notably, a substantial proportion of parents of unvaccinated daughters harboured concerns about the safety and efficacy of the domestic vaccine. Specifically, 80.8% of them were worried about serious side effects, 80.2% believed that important vaccines are safer and more effective than domestic ones, and 81.3% agreed that new vaccines carry more risks than traditional ones. These concerns help explain why parents with higher economic levels and previous vaccination experiences were more inclined to refuse the governmental vaccination services for their daughters. To effectively address vaccine hesitancy among parents of eligible girls, there is a pressing need for improved knowledge about HPV vaccines and access to reliable information sources [19]. However, the lasting impact of health education interventions remains uncertain. For instance, a randomised controlled trail in Japan demonstrated that while a cervical cancer survivor’s story initially increased parents’ willingness to consider HPV vaccination, this effect did not persist and failed to increase vaccination rates among children [30]. Echoing these findings, a recent systematic review by Escoffery et al. emphasised the need to broaden HPV vaccination promotion strategies beyond just educational efforts [31]. In this regard, the development and evaluation of comprehensive interventions that encompass multiple components and levels, targeting not only individual behaviours but also healthcare provider practices, are essential [32]. Future research should, therefore, focus on evaluating the effectiveness of these interventions in different contexts and populations.

### Strengths and limitations

This study boasts several significant strengths, particularly its comprehensive sampling approach. By drawing from a diverse population across various socioeconomic backgrounds in Shenzhen, the findings offer broad generalisability within the region. The employment of the validated VHS as a data collection tool adds credibility to the measurement of vaccine hesitancy among parents. Additionally, the timing of the study, conducted just prior to the start of the governmental HPV vaccination programme, offers crucial baseline data to shape future vaccination strategies. Moreover, the study’s multifaceted examination of factors like socio-economic status, insurance coverage, and parental vaccination history provides a comprehensive understanding of vaccine acceptance determinants.

However, the study is not without its limitations. The cross-sectional design restricts the ability to infer causality between the observed factors and vaccine hesitancy or acceptance. The distribution process of survey questionnaire, facilitated by the Municipal Education Bureau, limited direct access to the precise number of non-respondents and refusals, potentially affecting the assessment of the survey’s reach and representativeness of the sample. Furthermore, the adaptation of the original VHS to fit the local context and the removal of two items may have impacted the scale’s sensitivity and specificity in measuring vaccine hesitancy. The dependence on self-reported data raises the possibility of recall and social desirability biases. Geographically, while the study encompasses a diverse Shenzhen population, the results might not fully apply to other regions with distinct cultural and socio-economic contexts. Potential unmeasured confounders not accounted for in the study could influence the relationships between the examined factors and vaccine hesitancy or acceptance. Additionally, the lack of longitudinal follow-up prevents tracking changes in parental attitudes toward HPV vaccination, particularly in response to the government-funded programme.

## Conclusions

This study highlights the complex dynamics influencing parental attitudes towards HPV vaccination in Shenzhen, China. Despite low initial uptake among adolescent girls, there is a high willingness to accept the government-funded vaccines. Vaccine hesitancy, significantly influenced by safety concerns and a preference for imported vaccines, emerges as a major barrier to parental acceptance, even when controlling for various factors. The findings emphasise the need for scaling up HPV vaccination among eligible girls to align with WHO’s aim of vaccinating 90% of girls by the age of 15 by 2030. This process can be facilitated by the adoption of school-based vaccination programmes. Moreover, tailored communication strategies and comprehensive interventions beyond mere educational efforts are crucial to address the diverse concerns and socio-economic factors influencing vaccine acceptance. This approach is essential for enhancing HPV vaccine uptake and ultimately reducing the burden of cervical cancer.

### Electronic supplementary material

Below is the link to the electronic supplementary material.

Supplementary Material 1

## Data Availability

The survey data collected for this study are not publicly available due to concerns about participant confidentiality. Access to the data may be requested from the corresponding author on reasonable grounds, subject to approval by the Medical Ethics Committee of Shenzhen Maternity and Child Healthcare Hospital.

## Abbreviations

HPV: Human papillomavirus
WHO: World Health Organization
VHS: Vaccine Hesitancy Scale
SAS: Statistical Analysis Software
VIF: Variance inflation factor
OR: Odds ratio
CI: Confidence interval
