# Supplementary material for: Willingness and hesitancy towards the governmental free human papillomavirus vaccination among parents of eligible adolescent girls in Shenzhen, Southern China
Source: BMC Womens Health. 2024 Apr 23;24:253. doi: 10.1186/s12905-024-03083-2 (PMC11036762; doi:10.1186/s12905-024-03083-2)
Supplement: Supplementary file 1 — Supplementary Material 1 [file 12905_2024_3083_MOESM1_ESM.docx]

**Supplementary material**

**Questionnaire on Parents' Awareness of and Willingness to Vaccinate Eligible Girls against Human Papillomavirus (HPV)**

Dear parents, to gauge your awareness and willingness to vaccinate your school-age daughters against HPV, as well as to assess the current vaccination status in Shenzhen, and to better facilitate the free HPV vaccination initiative for school-age girls, we kindly ask for a few minutes of your time to complete this questionnaire. This questionnaire is anonymous, and all collected data will serve solely as a reference for improving HPV vaccination efforts among school-age girls. Please rest assured and fill in the questionnaire based on your actual situation. This questionnaire includes fill-in-the-blank, single-choice, and multiple-choice questions. We thank you in advance for your cooperation and valuable input.

**Sociodemographic information**

1. Your relationship with your daughter: (1) Mother-daughter (2) Father-daughter, (3) Others, please specify: ______________
2. Your daughter's school: ______________
3. Your daughter’s birthday: ______________
4. Your nationality: ① Chinese mainland; ② Hong Kong, Macao and Taiwan; ③ Foreign
5. Your medical insurance type (multiple choices allowed): ① Tier 1 Shenzhen Employee Medical Insurance; ② Tier 2 or Tier 3 Shenzhen Employee Medical Insurance; ③ Cross-regional medical insurance; ④ Commercial medical insurance; ⑤ Out-of-pocket payment; ⑥Others, please specify: ______________
6. Your daughter's medical insurance type (multiple choices are allowed): ① Shenzhen Children's Medical Insurance; ② Shenzhen Family Medical Insurance; ③ Cross-regional medical insurance; ④ Commercial medical insurance; ⑤ Out-of-pocket payment; ⑥Others, please specify: ______________
7. Do you have direct relatives diagnosed with cervical cancer? ① Yes; ② No; ③ Unsure

**Awareness and knowledge of HPV and HPV vaccine**

1. What is your opinion on HPV vaccine?

|  | Strongly agree | Somewhat agree | Somewhat disagree | Strongly disagree |
| --- | --- | --- | --- | --- |
| 1. Getting the HPV vaccine is a good way to protect my daughter from cervical cancer. |  |  |  |  |
| 1. Having my daughter vaccinated against HPV is important for the health of others. |  |  |  |  |
| 1. The HPV vaccine offered by the government programme is beneficial. |  |  |  |  |
| 1. The information l receive about the HPV vaccine from the vaccination programme is reliable. |  |  |  |  |
| 1. Generally, I do what healthcare providers recommend about vaccines for my daughter. |  |  |  |  |
| 1. New vaccines carry more risks than older vaccines. |  |  |  |  |
| 1. Domestic vaccines are less effective and safe compared to imported vaccines. |  |  |  |  |
| 1. l am concerned about serious side effects of the HPV vaccine. |  |  |  |  |

**Willingness to receive the free HPV vaccine**

1. Are you aware that Guangdong Province will soon offer free HPV vaccination for girls under the age of 15? ① Yes; ② No
2. If the government provides free HPV vaccination services for all girls in Grade 7 in the city, would you consent to the vaccination? ① Yes; ② No
3. If the government offers free domestic bivalent HPV vaccines for all girls in Grade 7 in the city, would you be willing to accept the vaccine for your daughter? ① Yes (skip to question 13); ② No
4. If unwilling, please specify the reason: ______________

**4. HPV vaccination behaviour**

1. Have you (the girl's mother) been vaccinated against HPV? ① Yes; ② No
2. Has your daughter (if you have more than one daughter, this question refers to the girl in the grade specified for this survey) been vaccinated against HPV? ① Yes; ② No (skip to 16)
3. Which HPV vaccine(s) did your daughter receive: ① domestic bivalent HPV vaccine; ② imported bivalent HPV vaccine; ③ imported quadrivalent HPV vaccine; ④ imported 9-valent HPV vaccine; ⑤ Unsure
4. Are there any other concerns or issues you have regarding the free HPV vaccination for girls in Grade 7? Please specify: ______________
